# Hydration of Cement in the Presence of Biocidal Modifiers Based on Metal Hydrosilicates

**DOI:** 10.3390/ma15010292

**Published:** 2021-12-31

**Authors:** Anna N. Grishina, Evgenij V. Korolev, Vitaliy A. Gladkikh

**Affiliations:** REC “Nanomaterials and Nanotechnology”, Research Center “MGSU Stroy-Test”, National Research Moscow State University of Civil Engineering, 129337 Moscow, Russia; korolev@nocnt.ru (E.V.K.); gladkikhVA@mgsu.ru (V.A.G.)

**Keywords:** composite cement binder, barium hydrosilicates, zinc hydrosilicates, copper hydrosilicates, hydration, differential thermal analysis, IR spectroscopy, setting time, strength

## Abstract

This article presents the results of a study of the characteristics of hydration and properties of a composite biocidal cement binder containing hydrosilicates of barium, copper or zinc. It was found that copper hydrosilicates block hydration processes, and when zinc hydrosilicates are used, the rate of hydration is determined by the content of silicic acid. The limiting concentrations of biocidal modifiers have been established: zinc hydrosilicates—no more than 4% and copper hydrosilicates—no more than 0.5%, which are advisable to use for the manufacture of a biocidal composite binder. It is shown that modifying additives slow down the setting time, the amount of tricalcium silicate in cement stones increases, and their strength for some compositions decreases. Active binding of portlandite with the formation of calcium hydrosilicates occurs when the content of zinc hydrosilicates is 2%, which leads to an increase in the strength of the materials.

## 1. Introduction

One of the promising directions in obtaining energy-efficient and environmentally friendly (helping to reduce the “carbon footprint”) building materials is the creation of composite binders through the use of modifying additives (industrial waste, specially synthesized modifiers obtained by low-temperature technologies) in the binder. These modifying additives include natural silicate additives—flasks, tripoli, diatomites, industrial waste—ash, microsilica, as well as synthetic silicate additives—metal hydrosilicates and other additives [1,2,3,4,5,6,7,8]. They are used in the composition of composite cements in order to reduce Portland cement in its composition while maintaining its strength characteristics or imparting specified properties. In this case, the choice of the modifier is determined by its ability to regulate several properties of the composite simultaneously [9]. Thus, the use of various silicate additives has already demonstrated its effectiveness in controlling the characteristics of cement composites [5,6,7,10]. One of the actual problems in civil and industrial construction is the destruction of materials under the influence of microorganisms, in particular, molds [11,12]. Features of the fight against biocorrosion of building materials include:

(1) surface treatment of structures with deep lesions;
(2) the use of organic biocides, which can degrade over time;
(3) the use of biocides, which are effective only in their pure form, and not in a concrete composition.

Therefore, a promising direction is the development of biocidal composite binders, which make it possible to provide long-term volumetric protection of the material from biocorrosion. In accordance with the data [13,14], the use of substances containing copper and zinc makes it possible to ensure the biocidal properties of cement composites, which helps to solve the problem of material destruction due to biocorrosion [15,16,17,18,19,20,21]. Thus, hydrosilicates of zinc and copper can be a promising additive for the creation of composite binders, since they allow reducing the consumption of cement in the binder and ensuring the biostability of the resulting cement composites. Previously, this approach to combating the formation of colonies of mold fungi in cement composites has not been used. The essence of the action of zinc and copper salts lies in their interaction with the waste products of molds—organic acids. As a result, Cu^2+^ and Zn^2+^ ions are formed, which can lead to the death of microorganisms. Thus, the biocide is activated by the waste products of molds and ceases to act when their activity is suppressed. Composite binders of this kind have not been developed before. According to [22], the copper ion is a more effective biocide than the zinc ion. However, it is known [22] that the hydration of cement in the presence of zinc- and copper-containing additives slows down, and zinc-containing additives more actively slow down hydration than copper-containing [19]. For practical application, this is a significant disadvantage, since it reduces the efficiency of construction production technology, namely, the timing of construction work increases. According to [10,23], in the presence of barium hydrosilicates, cement hydration is accelerated. Thus, presumably it is possible to regulate the processes of cement hydration. Studies of the joint effect of barium hydrosilicates and zinc or copper hydrosilicates on the processes of cement hydration and the properties of cement stone have not yet been carried out. Therefore, the purpose of the study is to obtain new knowledge about the effect of modifying additives on the rate of cement hydration in a composite biocidal binder, the peculiarities of the chemical composition of the resulting cement stones, the rate of structure formation by changes in the setting time and strength characteristics of the obtained cement stones. To establish the effect of the type and amount of biocidal modifying additives on the hydration of the composite biocidal binder, methods of IR spectroscopy and differential thermal analysis were used, the setting times were determined using a Vic’s device, and a servo-hydraulic system was used to determine the strength. The results obtained make it possible to identify factors that change the rate of cement hydration in the presence of hydrosilicates of zinc and copper, to establish their maximum rational and optimal concentrations in a composite biocidal binder, to give recommendations on the use of these binders in construction.

## 2. Materials and Methods

For the research, we used cement grade CEM I 42.5 N, corresponding to GOST 31108-2016. To create a biocidal composite binder, micro-sized barium hydrosilicates were introduced into Portland cement in an amount of 10% by weight of cement (changes in the strength of a composite binder, depending on the type and amount of barium hydrosilicates, were carried out and presented in [24]) and zinc hydrosilicates in an amount of 0.5, 1.0, 2.0, 3.0, 4.0, 5.0 and 6% or hydrosilicates of copper in an amount of 0.25, 0.5%, 0.75 and 1% by weight of cement. These modifiers were synthesized by the technology of hydrochemical synthesis from sodium hydrosilicates and aqueous solutions of salts—copper and barium chloride, as well as zinc sulfate. For the synthesis of metal hydrosilicates into a solution of sodium hydrosilicates with a silicate modulus *M*_Si_ = 3.0; ω = 26.5% and solutions of salts with concentrations of *C*(ZnSO**_4_**) = 12%; ***C***(CuCl**_2_**) = 15%; *C*(BaCl**_2_**) = 9.5%. The synthesis was carried out by adding a salt solution (ZnSO**_4_**, CuCl**_2_** or BaCl**_2_**) to a solution of sodium hydrosilicates at a MeO/SiO**_2_** ratio of 1.0. The resulting precipitate was thoroughly washed with distilled water, dried at a temperature of 100–105 °C and crushed. The average particle diameter of the obtained modifiers was 5–7 micrometers. The chemical composition of the modifying additive based on barium hydrosilicates additionally contains a small amount of barium carbonates formed during carbonization of the barium salt as a result of mixing during synthesis. A modifying additive based on zinc hydrosilicates additionally contains a small amount of Zn**_4_**SO**_4_** (OH)**_6_**xH**_2_**O. The chemical composition of modifying additives is presented in more detail in [25,26,27]. Cement, hydrosilicates of barium in an amount of 10% by weight of cement and hydrosilicates of zinc or copper were thoroughly mixed until homogeneous. A cement paste of normal density with water cement ratio (W/C) = 0.32 was made from the binder, and samples were formed, which were stored in a normal hardening chamber. Determination of the chemical composition of the samples was carried out on days 1, 3, 7, 14 and 28 of hardening. After testing the strength, the samples were crushed, after which the hydration of the binder was stopped. To stop hydration, the technique presented in [28] was used, which consists in microwave processing of the sample. The choice of a method for stopping hydration was dictated by the ability to quickly dehydrate the cement stone without the use of organic substances. This technique allows you to exclude foreign substances in the sample, and there is also a high speed of sample preparation. A sample for chemical analysis was taken by the quartering method from a dewatered cement stone powder obtained by grinding strength-tested samples. For chemical analysis of samples, we used differential thermal analysis methods using a HDSC PT1600/1400 high-temperature differential scanning calorimeter and a Cary 630 IR spectrometer. The sample heating rate in the calorimeter was 10 degree/min, the maximum heating temperature was 1000 °C. Thermograms were processed using HDSC evaluation software for reading and analyzing thermograms. The enthalpy value was estimated from the area of the anomaly between the differential curve and the baseline. For quantitative analysis, we used anomalies characteristic of the decomposition of one substance or for conditions of possible comparison of the content. When analyzing the effect of additives on the hydration of Portland cement by IR spectroscopy, all other things being equal, the method of comparing the intensities of characteristic peaks was used. An additive-free cement stone was used as a control composition. When comparing the values of enthalpies and the intensity of responses in the IR spectra, the proportion of Portland cement in the original binder was taken into account. The setting time was determined using a Vic device in accordance with GOST 30744-2001 “Cements. Test methods using polyfraction sand”. The compressive strength was determined on cubic specimens with an edge of 20 mm. To determine the strength, a servo-hydraulic system for static and low-frequency dynamic tests of building materials ADVANTEST 9 was used. A series of 15 samples was used for testing. Based on the results obtained, the mean value and statistical indicators were calculated: the standard deviation and the coefficient of variation, which for strength did not exceed the standard value,13%. The relative error of physical measurements did not exceed 5%.

## 3. Results and Discussion

**Figure 1:**
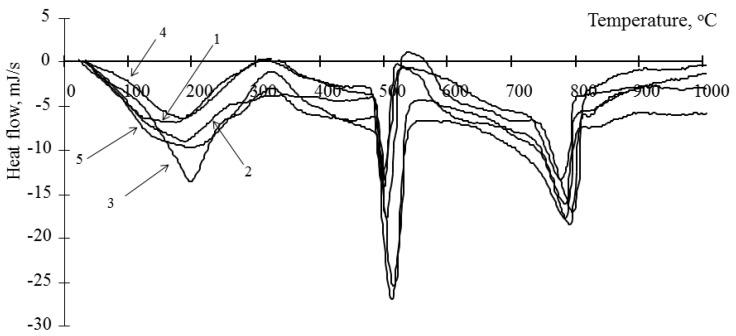
Thermogram of a cement stone modified with barium hydrosilicates and zinc hydrosilicates (0.5%): 1—at the age of 1 day; 2—at the age of 3 days; 3—at the age of 7 days; 4—at the age of 14 days; 5—at the age of 28 days.

**Figure 2:**
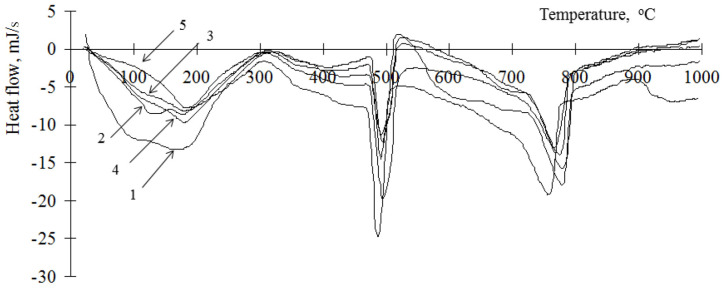
Thermogram of a cement stone modified with barium hydrosilicates and copper hydrosilicates (0.5%): 1—at the age of 1 day; 2—at the age of 3 days; 3—at the age of 7 days; 4—at the age of 14 days; 5—at the age of 28 days.

Analysis of the hydration processes of composite binders can be carried out on the basis of a quantitative analysis of the resulting products. For this, the enthalpies of the anomalies on the obtained thermograms were determined. The enthalpies were grouped according to the temperature range of the minimum points. For the analysis, anomalies were selected that correspond to the decomposition of portlandite (480–500 °C) [29,30] and hydrosilicates and calcium carbonate (750–780 °C) [29,30]. Typical thermograms of a cement stone modified with hydrosilicates of the studied metals (barium, zinc, copper), using the example of a cement stone modified with hydrosilicates of zinc and copper in an amount of 0.5%, are shown in Figure 1 and Figure 2. The results of determining the enthalpies for anomalies are presented in Table 1.

Considering that portlandite is formed during the hydration of the main clinker minerals—tricalcium silicate (C_3_S) and dicalcium silicate (C_2_S)—its content can be used to estimate the degree of hydration of the cement binder, taking into account the proportion of cement in it. The content of portlandite in the material can be estimated from the change in the anomaly at 450–500 °C [29]. It should be borne in mind that it can increase with an increase in the degree of hydration of cement and decrease due to the binding of portlandite by amorphous hydrosilicates of metals. This anomaly characterizes the decomposition of only one mineral; therefore, it is most convenient for the analysis of hydration processes. Decomposition of hydrosilicates and calcium carbonate occurs at a temperature close to the value 750–780 °C [29]. Considering that carbonate is formed only in the surface layer of the samples and during sample preparation, this anomaly can be indirectly used to estimate the content of calcium hydrosilicates. Analyzing the change in the content of portlandite in the cement stone, it should be noted that its concentration in the cement stones modified by hydrosilicates of copper is lower than in the control composition (Table 1). At the same time, a decrease in its content is observed with an increase in the concentration of copper hydrosilicates. Therefore, with the content of copper hydrosilicates in the amount of 1% at the age of 1 day, portlandite was not found in the sample. When using hydrosilicates of zinc on the 1st day of hydration, the concentration of portlandite is also lower than for the control composition (from 23 to 84%) and is determined by the content of the modifier. At the same time, on the 28th day of hydration, the portlandite concentration can be either below the control value or exceed the control values (up to 121%) with a zinc hydrosilicate content of 2–3%. Thus, the concentration of portlandite in the initial period of hardening decreases when both types of modifiers are used. In this case, the concentration of the additive has a significant effect. Thus, an increase in the content of copper hydrosilicates in the binder has a strong “toxic” effect on cement in the initial period of hydration, which is indicated by a significant decrease in the concentration of portlandite, and hydrosilicates of zinc, depending on the concentration, can both accelerate and slow down the rate of hydration of the main clinker minerals. In addition, it should be noted that, in contrast to the control composition, the kinetics of changes in the concentration of portlandite in the modified samples has a complex vibrational character (Table 2). This can be explained as follows. It is known that portlandite is able to enter into chemical interaction with amorphous hydrosilicates, while the formation of calcium hydrosilicates is possible. A similar oscillatory nature of changes in the calcium concentration in systems containing silicic acid is noted in [31]. Binding of portlandite is a constructive process that makes it possible to increase the strength of the resulting stone, as well as an initiator of the hydration of C_3_S and C_2_S (except for the cases of the formation of dense impermeable shells on the surface of mineral particles). It is possible to indirectly estimate the concentration of calcium hydrosilicates by the value of the enthalpy at 750–780 °C (Table 1). Thus, at a concentration of zinc hydrosilicates of 6% and copper hydrosilicates of 1%, the content of calcium hydrosilicates during the entire studied period of hydration is less than in the control composition (94% compared to the control composition). This indicates the lack of binding of portlandite, thus the low concentration of portlandite is due to the low rate of hydration of C_3_S and C_2_S. For other compositions, the concentration of calcium hydrosilicates exceeds the control values at the age of 28 days—up to 220% when using hydrosilicates of zinc and up to 270% when using hydrosilicates of copper, while the values of the control composition are exceeded after 3 days of hardening. Thus, the complex oscillatory nature of changes in the concentration of portlandite is due to its binding to calcium hydrosilicates. The reduction in portlandite in the resulting cement stone is beneficial in reducing the likelihood of leaching processes. Thus, in works [10,32] the possibility of a significant decrease in the content of portlandite in the composition of the artificial stones obtained is shown.

**Figure 3:**
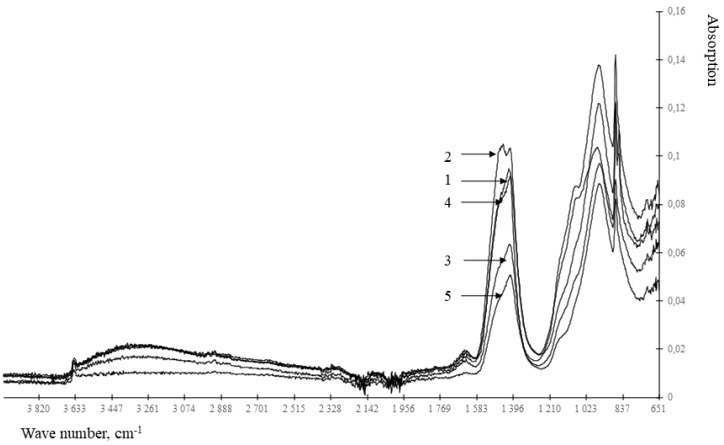
IR spectra of cement stone modified with barium hydrosilicates and copper hydrosilicates (0.5%): 1—at the age of 1 day; 2—at the age of 3 days; 3—at the age of 7 days; 4—at the age of 14 days; 5—at the age of 28 days.

**Figure 4:**
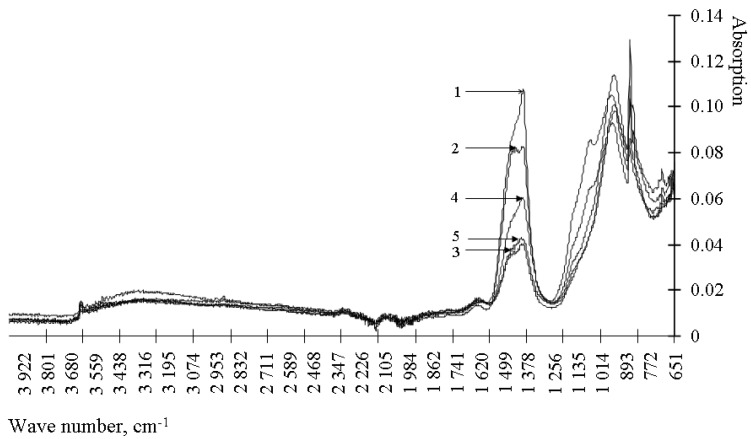
IR spectra of cement stone modified with barium hydrosilicates and zinc hydrosilicates (0.5%): 1—at the age of 1 day; 2—at the age of 3 days; 3—at the age of 7 days; 4—at the age of 14 days; 5—at the age of 28 days.

Taking into account the disadvantages of the method of differential thermal analysis, namely, the difficulty in identifying clinker minerals, as well as to establish a compound that has a blocking effect on the hydration of clinker minerals, the method of IR spectroscopy was used. A typical IR spectrum of a cement stone modified with metal hydrosilicates is shown in Figure 3 and Figure 4 and in Table 2.

Analysis of the results presented in Figure 3 and Figure 4 and Table 2 shows that the control composition and the compositions modified with hydrosilicates of zinc differ in the absence of a spectrum at 1163–1167 cm^−1^. The specified spectrum corresponds to the S-O fluctuations, that is, it determines the content of the gypsum stone. According to the Table 2 in the presence of zinc hydrosilicates, the gypsum stone is bound with the formation of ettringite already after 1 day of hardening. When using hydrosilicates of copper, even after 28 days, there is no complete binding of the gypsum stone. Zinc hydrosilicates do not slow down the formation of ettringite—the gypsum stone is bound after 1 day of hardening. Similar conclusions can be drawn from the change in the intensity of the spectrum at 940–965 cm^−1^, corresponding to C_3_S. When using hydrosilicates of copper, the content of C_3_S in the cement stone is 1.45–2.43 times higher than its content in the control composition after 1 day of hardening, which is comparable with the use of of hydrosilicates of zinc. However, after 28 days of hardening, the C_3_S content exceeds the control values by 2–17% when using hydrosilicates of copper, and when using hydrosilicates of zinc, it can either decrease to 7% (with a concentration of zinc hydrosilicates of 2%) in comparison with the control composition, or exceed the control value by ~1.8 times (at a concentration of zinc hydrosilicates 5–6%). The presented data confirm the complex nature of the influence of zinc hydrosilicates on the hydration processes of C_3_S, namely, the acceleration of hydration at a concentration of 2–3%. Additionally, it should be borne in mind that responses in the range of 800–1100 cm^−1^ are also characteristic of calcium hydrosilicates, therefore, an increase in the signal at 940–965 cm^−1^ may indicate the formation of calcium hydrosilicates. Thus, when using hydrosilicates of copper, the rate of hydration is determined by the copper content of the modifier. When using hydrosilicates of zinc, the rate of hydration is probably determined by the concentration of silicic acid in the test material, the concentration of zinc ions increases linearly, and the change in the amount of C_3_S occurs nonlinearly. Such changes are manifested when using silicic acids [33,34] in the composition of cement composites, and they can be traced by changing the setting time of the composite cement paste. The results are shown in Table 3.

According to the Table 4, the Zn^2+^ and Cu^2+^ ions have a significantly different effect on the earlier structure formation in the binder. Thus, the accelerating effect of barium hydrosilicates is completely blocked by the introduction of even a small amount of hydrosilicates of copper. The retarding effect increases with an increase in the content of the modifying additive and at a concentration of copper hydrosilicates of 1%, the composite binder does not meet the requirements of regulatory documents at the end of setting (the end of setting is not later than 10 h of hardening). End-of-set time also increases with hydrosilicates of copper at all tested concentrations. This indicates a slowdown in the processes of cement hydration with copper ions, which is consistent with the data in Table 1 and Table 2 and Figure 3. The amount of silicic acid introduced into the material together with hydrosilicates of copper has a weak effect on hydration. Therefore, some acceleration of the beginning (0.75% of hydrosilicates of copper) and the end (0.5% of hydrosilicates of copper) setting is caused by the features of depolymerization of silicic acid, which is contained in modifiers of hydrosilicates of copper and hydrosilicates of barium. Silicic acid and silicates, depending on the pH values of the solution, the concentration, change the rate of depolymerization [35,36] and, accordingly, the ability to actively adsorb calcium ions from the solution. When using hydrosilicates of zinc, the modifier weakly changes the properties of the material, that is, zinc ions, unlike copper, do not lead to a rapid slowdown in the hydration of cement. Barium ions and silicic acid accelerate the onset of setting. With an increase in the content of zinc hydrosilicates to 1%, the content of the silicate phase increases, and the depolymerization conditions are maintained, which accelerates the onset of setting, while zinc ions also do not prevent the acceleration of hydration processes. When the content of zinc hydrosilicates is 2–3%, there is a slight slowdown in the depolymerization of silicic acid, and then acceleration again, which is reflected in the timing of the onset of setting. With an increase in the content of zinc ions, the setting time has a non-linear character, therefore, in general, it is the concentration of silicic acid, and not the concentration of zinc, that has a dominant effect. Therefore, in the presence of zinc hydrosilicates, blocking of gypsum binding does not occur. It should be noted that the data obtained contradict the known concepts [22] about a more active retardation of cement hydration by zinc in comparison with copper. Therefore, when choosing a retarder, one should take into account the anion of the salt containing copper or zinc.

**Figure 5:**
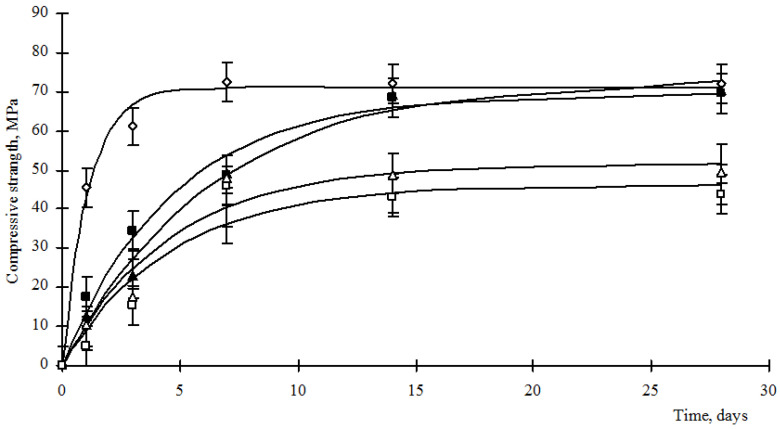
Strength of cement stone modified with barium hydrosilicates and copper hydrosilicates, where: ◊—control composition; ■—content of hydrosilicates of copper 0.25%; ▲—content of hydrosilicates of copper 0.50%; ∆—content of hydrosilicates of copper 0.75%; □—content of hydrosilicates of copper 1.0%.

**Figure 6:**
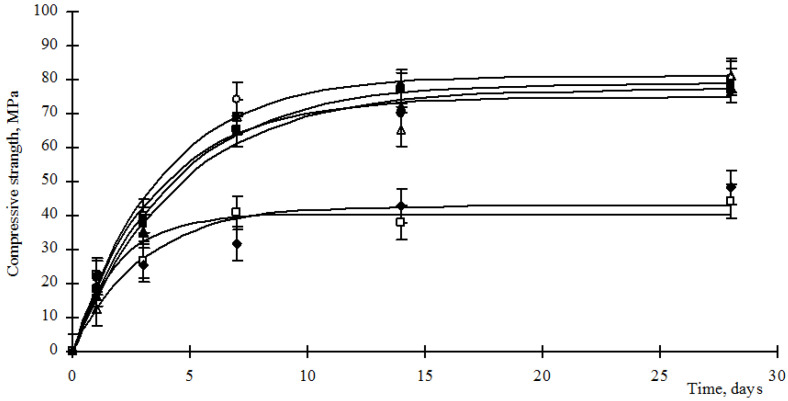
Strength of cement stone modified with barium hydrosilicates and zinc hydrosilicates, where: ○—content of hydrosilicates of zinc 0.5%; ●—content of hydrosilicates of zinc 1%; ■—content of hydrosilicates of zinc 2%; ▲—content of hydrosilicates of zinc 3%; ∆—content of hydrosilicates of zinc 4%; □—content of hydrosilicates of zinc 5%; ♦—content of hydrosilicates of zinc 6%.

Thus, the introduction of hydrosilicates of metals into Portland cement can both initiate structural processes and slow them down, which affects the properties of the resulting cement stones. Since when creating a composite binder, it is necessary to preserve the grade for the strength of the cement used, then the strength of the resulting stones should be established. In order to formulate recommendations for the use of concrete mixtures on construction sites, the kinetics of the strength gain of the materials should be established. The results of strength gain with composite cement stones are shown in Figure 5 and Figure 6.

Analysis of Figure 5 and Figure 6 shows that the strength gain of composite cement stones is carried out according to the exponential law (1):

*R_st_* = *R_max_*(1 − e*^−^^bt^*), (1)

where *R_st_* is the compressive strength of the cement stone, MPa; *R_max_*—maximum strength of cement stone, MPa; *b* is an empirical coefficient characterizing the rate of strength gain; *t*—time, day. The values of the empirical coefficients are given in Table 4.

Analysis of the data in Figure 5 and Figure 6 and Table 4 shows that the use of hydrosilicates of copper in an amount of 0.25–0.5%, as well as hydrosilicates of zinc in an amount of not more than 4%, allows achieving the strength of a control cement stone at a vintage age with a lower cement consumption. However, the development of strength by such materials proceeds more slowly, as indicated by a decrease in the values of the coefficient b (Table 3). The use of hydrosilicates of zinc in an amount of 5–6% increases the rate of strength development, but the *R_max_* value decreases significantly.

Comparing the data in Table 1, Table 2, Table 3 and Table 4, we can make the following conclusion. Considering that, according to the thermograms, the content of calcium hydrosilicates in modified cement stones is comparable to or exceeds the values for the control composition, as well as a low content of portlandite and a high content of C_3_S, the formation of calcium hydrosilicates is carried out by interaction of portlandite with the amorphous part of hydrosilicates of barium, zinc and copper. This takes place when using hydrosilicates of copper in an amount of 0.25–0.5%, as well as hydrosilicates of zinc in an amount of not more than 4%. The formed hydrosilicates of calcium are formed more slowly than in the control composition. However, their amount is sufficient to compact the structure of the cement stone, which ensures the achievement of the required strength or even exceeding the strength values of the control composition. With an increase in the concentration of hydrosilicates of copper more than 0.5% and hydrosilicates of zinc more than 4%, the content of copper ions and silicates reaches a critical value, blocking the hydration of C_3_S and C_2_S. Despite the fact that the binding of portlandite should initiate the hydration of C_3_S and C_2_S, the negative effect of copper ions and blocking of the C_3_S surface by hydration products is dominant. Thus, the resulting cement stone has a large supply of clinker stock, which is promising for ensuring the healing of microcracks arising during the operation of structures made of cement concrete. When manufacturing products from composite cement, one should consider the low rate of hydration of the binder and prevent its premature loading.

## 4. Conclusions

1. It was found that in a biocidal composite binder hydrosilicates of copper have a significant retarding effect on the processes of hydration of cement than hydrosilicates of zinc. The effect of zinc hydrosilicates on hydration is determined by the content of the silicate phase; therefore, hydration can both accelerate and slow down.

2. The limiting concentrations of biodiceous modifying additives have been established: zinc hydrosilicates—no more than 4% and copper hydrosilicates—no more than 0.5%, which allow to obtain equal strength or more durable cement stone from a composite binder with a reduced cement content.

3. The content of calcium hydrosilicates in the formed cement stones exceeds or is comparable to its content in the control cement stone, subject to the recommended dosages. This is due to the occurrence of processes of binding of portlandite by the silicate phase of hydrosilicates of barium, copper and zinc. Reducing the amount of portlandite is an advantage in providing improved leach resistance.

## Figures and Tables

**Table 1:** Values of enthalpy.

Enthalpy, J/g for the Response with Extremum, °C	Age, day				
	1	3	7	14	28
0.5% of hydrosilicate zinc					
480–500	−21.19	−31.51	−41.43	−36.99	−40.95
750–780	−31.74	−45.84	−31.43	−55.30	−42.76
1.0% of hydrosilicate zinc					
480–500	−18.34	−15.62	−38.73	−28.78	−37.55
750–780	−28.67	−39.74	−49.54	−51.96	−49.61
2.0% of hydrosilicate zinc					
480–500	−28.04	−36.85	−42.89	−48.37	−58.81
750–780	−41.93	−54.80	−39.70	−51.62	−49.52
3.0%of hydrosilicate zinc					
480–500	−26.15	−32.62	−42.62	−44.60	−51.12
750–780	−32.89	−45.82	−58.74	−54.30	−56.02
4.0% of hydrosilicate zinc					
480–500	−24.04	−37.07	−34.04	−45.20	−31.28
750–780	−29.51	−42.29	−44.29	−46.45	−41.48
5.0% of hydrosilicate zinc					
480–500	−10.60	−18.12	−20.97	−18.01	−21.16
750–780	−18.82	−27.66	−31.05	−29.97	−29.22
6.0% of hydrosilicate zinc					
480–500	−7.30	−17.37	−10.72	−16.94	−15.17
750–780	−12.04	−25.01	−26.03	−25.85	−23.26
0.25% of hydrosilicate copper					
480–500	−28.99	−31.04	−31.06	−49.09	−43.22
750–780	−32.88	−43.02	−45.97	−48.52	−40.33
0.5% of hydrosilicate copper					
480–500	−29.23	−29.91	−32.37	−31.23	−48.21
750–780	−39.39	−46.16	−43.38	−49.24	−44.00
0.75% of hydrosilicate copper					
480–500	−26.45	−30.22	−31.63	−44.98	−45.41
750–780	−42.77	−49.18	−50.33	−60.58	−69.00
1% of hydrosilicate copper					
480–500	–	−5.46	−13.76	−21.86	−25.02
750–780	−2.75	−13.33	−24.11	−26.24	−25.11
Control composition					
480–500	−37.22	−45.83	−53.92	−54.32	−54.43
750–780	−38.77	−44.00	−31.90	−29.15	−28.54

**Table 2:** Intensity of IR spectrum.

Wavenumber, cm^−1^	Age, days				
	1	3	7	14	28
0.5% of hydrosilicate zinc					
1163–1167	–	–	–	–	–
940–965	0.059549	0.090966	0.058821	0.072629	0.062233
1.0% of hydrosilicate zinc					
1163–1167	–	–	–	–	–
940–965	0.063768	0.074353	0.064744	0.079351	0.075371
2.0% of hydrosilicate zinc					
1163–1167	–	–	–	–	–
940–965	0.09096	0.05726	0.082424	0.055943	0.050913
3.0% of hydrosilicate zinc					
1163–1167	–	–	–	–	–
940–965	0.08281	0.070537	0.07114	0.066105	0.056942
4.0% of hydrosilicate zinc					
1163–1167	–	–	–	–	–
940–965	0.073808	0.089035	0.05995	0.064385	0.071096
5.0% of hydrosilicate zinc					
1163–1167	–	–	–	–	–
940–965	0.07302	0.068688	0.073117	0.067266	0.098813
6.0% of hydrosilicate zinc					
1163–1167	–	–	–	–	–
940–965	0.069865	0.07047	0.066473	0.065942	0.097897
0.25% of hydrosilicate copper					
1163–1167	0.015373	0.019754	0.019991		0.006881
940–965	0.058528	0.08853	0.07156	0.080415	0.056018
0.5% of hydrosilicate copper					
1163–1167	0.074429	0.022087	0.011307	0.011788	0.007657
940–965	0.087733	0.059298	0.060316	0.076183	0.060625
0.75% of hydrosilicate copper					
1163–1167	0.07637	0.023699	0.010411	0.004507	0.006494
940–965	0.082416	0.053709	0.095245	0.045252	0.062908
1.0% of hydrosilicate copper					
1163–1167	0.074429	0.02336	0.020879	0.012401	0.008841
940–965	0.0982229	0.065899	0.069552	0.058769	0.06402
Control composition					
1163–1167	–	–	–	–	–
940–965	0.040411	0.04763	0.038303	0.058102	0.054476

**Table 3:** Setting time of composite cement paste.

Modifier Type	Modifier Content, %	Setting Start Time, Hours: Min	End of Setting Time, Hours: Min	Duration of Setting, Hours: Min
Control composition		2:10	3:45	1:35
Copper hydrosilicates	0.25	2:25	4:35	2:10
	0.5	2:40	4:20	1:40
	0.75	2:20	5:45	3:25
	1.0	6:04	more than 10 h	more than 10 h
Zinc hydrosilicates	0.5	1:30	4:25	2:55
	1.0	0:55	4:00	3:05
	2.0	2:30	6:00	3:30
	3.0	2:30	3:35	1:05
	4.0	2:10	3:25	1:15
	5.0	1:05	3:10	2:05
	6.0	1:45	3:45	2:00

**Table 4:** Empirical coefficient values.

Modifier Type	Modifier Content, %	Empirical Coefficient Values	
		*R_max_*	*b*
Copper hydrosilicates	control composition	70.94	0.929
	0.25	69.68	0.212
	0.50	73.68	0.154
	0.75	51.64	0.218
	1.0	46.37	0.217
Zinc hydrosilicates	0.50	81.23	0.270
	1.0	75.09	0.277
	2.0	79.29	0.234
	3.0	77.67	0.222
	4.0	77.60	0.223
	5.0	40.49	0.538
	6.0	43.06	0.343

## Data Availability

Not applicable.
